# *Acinetobacter baumannii* infection in critically ill patients with COVID-19 from Tehran, Iran: the prevalence, antimicrobial resistance patterns and molecular characteristics of isolates

**DOI:** 10.3389/fcimb.2024.1511122

**Published:** 2025-01-30

**Authors:** Mahsa Ghamari, Fereshteh Jabalameli, Shirin Afhami, Shahnaz Halimi, Mohammad Emaneini, Reza Beigverdi

**Affiliations:** ^1^ Department of Microbiology, School of Medicine, Tehran University of Medical Sciences, Tehran, Iran; ^2^ Department of Infectious Diseases, Shariati Hospital, Tehran University of Medical Sciences, Tehran, Iran

**Keywords:** *Acinetobacter baumannii*, SARS-CoV-2, co-infection, antibiotic resistance, resistance genes, global clones, ICU, REP-PCR

## Abstract

**Background:**

The COVID-19 pandemic has led to the excessive use of antimicrobials in critically ill patients. Infections caused by *Acinetobacter baumannii* have increased significantly both regionally and globally during the COVID-19 pandemic, posing dramatic challenges for intensive care unit (ICU) patients. This study aimed to determine the prevalence, antimicrobial resistance patterns, presence of selected antimicrobial resistance genes, and genetic diversity of *A. baumannii* isolates obtained from COVID-19 cases admitted to the ICU at the University Hospital in Iran.

**Materials and methods:**

This was a cross-sectional and single-center study comprising patients with *A. baumannii* infections admitted to the ICU with COVID-19 between April and November 2021. The demographic and clinical data of the patients were collected. Antimicrobial susceptibility testing was conducted based on Clinical Laboratory Standards Institute guidelines. This study used PCR and multiplex PCR to investigate antibiotic resistance genes (ARGs) and global clones (GC), respectively. Genetic diversity was investigated by repetitive element sequence-based PCR (REP-PCR).

**Results:**

The prevalence of *A. baumannii* coinfection in COVID-19 cases was 8.1% (43/528). More than 90% (39/43) of *A. baumannii* isolates were resistant to cefepime, ampicillin-sulbactam, gentamicin, trimethoprim-sulfamethoxazole and amikacin. Furthermore, 44.2% (19/43) of isolates were resistant to colistin. There were 91% (39/43) isolates that were extensively drug-resistant (XDR). The most prevalence carbapenem resistance encoding genes were bla*_-OXA-23_* 65.1% (29/43) and bla*_NDM_* 41.8% (18/43). The most common aminoglycoside resistance genes were *aac(6’)-Ib* 65.1% (28/43) and *ant(2)-Ia* 46.5% (20/43). Isolates from the prominent Global clone GCII comprised 83.7% (36/43) of total isolates. Genetic fingerprinting using REP-PCR revealed that 39 typeable *A. baumannii* isolates were categorized into 12 distinct genotypes, of which 72% (28/39) of isolates belonged to one genotype.

**Conclusion:**

The high prevalence of XDR *A. baumannii* such as carbapenem and colistin-resistant strains, poses a significant concern for the treatment of COVID-19 patients, heightening the risk of therapeutic failure. The data demonstrate the dissemination of a single *A. baumannii* clone carrying multiple ARGs within our hospital. Regarding the limited therapeutic options, it is crucial to implement effective prevention and containment policies to curb the spread of these strains.

## Introduction

Respiratory infections caused by acute coronavirus syndrome 2 (SARS-COV-2) were first reported in December 2019 in Wuhan, China, and subsequently rapidly spread globally (Sharma et al., 2020). According to the World Health Organization (WHO) report dated December 30, 2023, the virus has been identified in more than 773 million individuals across 220 countries and has unfortunately caused the death of more than 7 million people (Organization WH, 2023). The clinical manifestations of coronavirus infection vary from asymptomatic infection to severe viral pneumonia, requiring treatment in an intensive care unit (ICU) or mechanical ventilation (Zeng et al., 2021). Hospitalization of COVID-19 patients, especially in the ICU, creates ideal conditions for acquiring healthcare-associated infections (HAIs) or secondary infections, particularly with multidrug-resistant (MDR) Gram-negative pathogens such as *Acinetobacter baumannii* (Ceparano et al., 2022). During the COVID-19 pandemic, there has been a significant increase in the prevalence of *A. baumannii* infections both regionally and globally (Boral et al., 2022; Mobarak-Qamsari et al., 2023; Petazzoni et al., 2023). This organism exhibits high rates of resistance to multiple antibiotics, including carbapenems, aminoglycosides, fluoroquinolones, and polymyxins, due to both rapidly acquired resistance genes and intrinsic resistance (Kyriakidis et al., 2021). In 2017, Carbapenem-resistant *A. baumannii* (CRAB) was listed at the top of WHO priorities for the development of new antibiotics (Tacconelli et al., 2018). Clonal spread of CRAB has been reported worldwide, with global clones 1 and 2 (GC1 and GC2) frequently associated with the increased resistance of this microorganism (Zarrilli et al., 2012). Infections caused by extensively drug-resistant (XDR) or MDR bacteria lead to higher ICU mortality and morbidity rates, along with increased healthcare costs, while available therapeutic options are limited (Barbato et al., 2019). The treatment of patients with COVID-19 co-infected with antibiotic-resistant bacteria circulating frequently in hospitals is extremely challenging. Therefore, in order to combat antibiotic resistance and implement prevention control practices, the detection of antibiotic-resistant bacteria is essential (Lucien et al., 2021). The aim of the current study was to evaluate the prevalence, antibiotic resistance profiles and genetic relatedness of *A. baumannii* isolates obtained from patients with COVID-19 infection admitted to the ICU in a tertiary care hospital in Tehran, Iran.

## Methods

### Setting and sampling

This was a cross-sectional and single-center study, including patients admitted to Shariati Hospital, a tertiary referral hospital in Tehran, Iran, between April and November 2021. Patients were included in the current study based on the following criteria (Sharma et al., 2020): laboratory-confirmed SARS-CoV-2 infection using an RT–PCR test on a nasopharyngeal swab (Organization WH, 2023); unilateral or bilateral interstitial infiltrates confirmed by chest X-ray (Zeng et al., 2021); the presence of acute hypoxemic respiratory failure requiring mechanical ventilation. Infections were considered nosocomial if the positive culture for *A. baumannii* was obtained more than 48 hours after hospital admission. To distinguish true co-infection from colonization, a combination of clinical, microbiological, and diagnostic criteria was employed. Only patients with positive cultures from both clinically relevant sites (e.g., blood, sputum, bronchoalveolar lavage (BAL), urine, wound) accompanied by signs and symptoms of infection, such as fever, respiratory distress, or sepsis, were considered to have co-infections. In contrast, patients with positive cultures from non-infectious sites (e.g., throat or rectal swabs) who did not exhibit clinical signs of infection were classified as colonized. Patient data were obtained from hospital computerized databases. The following information was collected: clinical, demographic, and laboratory findings; underlying conditions; microbiological data; duration of ICU stay.

### Definitions

Pulmonary infections were classified into two categories: (a) co-infections, referring to cases where patients with confirmed COVID-19 simultaneously harbored other pathogens within the first 48 hours, and (b) secondary infections, characterized by the emergence of new pathogenic infections occurring beyond 48 hours following hospital admission (Lansbury et al., 2020). A bloodstream infection is identified by the presence of a likely pathogen in at least one positive blood culture. For organisms common to skin flora, multiple positive cultures (e.g., two or more) are often needed to confirm infection and rule out contamination (Timsit et al., 2020). Septic shock was defined according to the 2016 Third International Consensus Definitions for Sepsis and Septic Shock (Singer et al., 2016). Urinary tract infection was diagnosed based on both clinical and microbiological evidences (Van Laethem et al., 2021).

### Isolate identification

One isolate per patient was included in this study. All bacterial isolates were identified using standard laboratory methods, including Gram-staining, oxidase and catalase tests, oxidative/fermentative tests, motility, and growth ability at 42°C (Vasconcellos et al., 2017). Species identification was confirmed by amplification of *gltA* gene (encoding species citrate synthase), as described previously (Wong et al., 2014). The *A. baumannii* isolates were stored at –70°C in trypticase soy broth with 20% glycerol until used for the study.

### Antibiotic susceptibility testing

The antimicrobial susceptibility was determined using the Kirby Bauer (disc agar diffusion) method based on Clinical and Laboratory Standards Institute (CLSI) guidelines (Weinstein MP et al., 2022). For disk diffusion test, eleven antimicrobial disks (MAST, United Kingdom) were used including: amikacin)30 μg(, gentamicin (10 μg), cefepime (30 μg), ceftazidime (30 μg), cefotaxime (30 μg), ciprofloxacin (5μg), piperacillin-tazobactam (10 μg), trimethoprim-sulfamethoxazole (30 μg), ampicillin-sulbactam (30μg), meropenem (10 μg) and imipenem (10 μg). The broth microdilution method was implemented to determine the minimum inhibitory concentration (MIC) of colistin according to CLSI guidelines (Weinstein MP et al., 2022). Additionally, the broth disk elution method was used to confirm the resistance to colistin according to CLSI guideline *Escherichia coli* ATCC 25922 and *Pseudomonas aeruginosa* ATCC 27853 were used as quality control strains. The investigated isolates which were resistant to three or more different classes of antibiotics except for carbapenems, were considered as MDR phenotype and MDR isolates which were resistant to meropenem, were considered as XDR phenotype (Kyriakidis et al., 2021).

### DNA extraction

To obtain genomic DNA, the boiling method was implemented as previously described (Chen et al., 2020). DNA purity and quantification was assessed using NanoDrop device (Thermo Scientific,Wilmington, DE, USA). The supernatant was stored at −20°C as the DNA template to be used in PCR reactions.

### Detection of antibiotic resistance genes

The presence of class D carbapenemases genes (*bla*_OXA−23−like_, *bla*_OXA−24−like_ and *bla*_OXA−58−like_) was investigated by multiplex-PCR method as described previously (Woodford et al., 2006). Furthermore, isolates harbouring the class B carbapenemases or metallobeta-lactamases (MBL) genes (*bla*_NDM_, *bla*_IMP_, *bla*_VIM-1_), class A carbapenemase (*bla*_KPC_, *bla*_TEM),_ aminoglycoside resistance genes (*aac(6′)-Ib, aac(3)-Ia, ant(2″)-Ia* and *aph(3’)- Ia*) and colistin resistance determinant (*mcr-1*) were detected by single PCR as described previously (Giakkoupi et al., 2003; Robicsek et al., 2006; Nguyen et al., 2010; Chen and Huang, 2013; Bina et al., 2015; Ghotaslou et al., 2017; Chen et al., 2020; Abdollahi et al., 2021; Pourajam et al., 2022). Positive and negative controls were used for each of the investigated ARGs during PCR. The primers and the PCR conditions used for the amplification are listed in **Table 1**.

**Table 1:** The primers and the conditions used for the amplification of genes encoding antibiotic resistance.

Gene	Primer sequence (5’ to 3’)	Product size (bp)	PCR programme*	Reference
***aac(6′)-Ib***	TTGCGATGCTCTATGAGTGGCTA CTCGAATGCCTGGCGTGTTT	482	94°C, 60s; 61°C, 40s; 72°C, 30s: 30 cycles	(Vasconcellos et al., 2017)
***aac(3)-Ia***	GCAGTCGCCCCTAAAACAAA CACTTCTTCCCGTATGCCCAACTT	464	94°C, 60s; 61°C, 40s; 72°C, 30s: 30 cycles	(Wong et al., 2014)
***ant(2″)-Ia***	ACGCCGTGGGTCGATGTTTGATGT CTTTTCCGCCCCGAGTGAGGTG	572	94°C, 60s; 67°C, 40s; 72°C, 35s: 30 cycles	
***aph(3’) Ia***	CGAGCATCAAATGAAACTGC GCGTTGCCAATGATGTTACAG	624	94°C, 60s; 57°C, 40s; 72°C, 30s: 30 cycles	
***TEM***	TGCGGTATTATCCCGTGTTG TCGTCGTTTGGTATGGCTTC	297	94°C, 60s; 55°C, 40s; 72°C, 30s: 30 cycles	(Weinstein MP et al., 2022)
***VIM-1***	AGTGGTGAGTATCCGACAG ATGAAAGTGCGTGGAGAC	261	94°C, 60s; 56°C, 40s; 72°C, 30s: 30 cycles	(Chen et al., 2020)
***KPC***	CGTCTAGTTCTGCTGTCTTG CTTGTCATCCTTGTTAGGCG	798	94°C, 60s; 60°C, 40s; 72°C, 40: 30 cycles	(Woodford et al., 2006)
***IMP***	CATGGTTTGGTGGTTCTTGT ATAATTTGGCGGACTTTGGC	448	94°C, 60s; 56°C, 40s; 72°C, 30s: 30 cycles	(Abdollahi et al., 2021)
***NDM***	GGTTTGGCGATCTGGTTTTC CGGAATGGCTCATCACGATC	621	94°C, 60s; 62°C, 40s; 72°C, 35s: 30 cycles	(Bina et al., 2015)
***mcr-1***	CGGTCAGTCCGTTTGTTC CTTGGTCGGTCTGTAGGG	309	94°C, 60s; 56°C, 40s; 72°C, 30s: 30 cycles	(Chen and Huang, 2013)
***OXA_-23-like_***	GATCGGATTGGAGAACCAGA ATTTCTGACCGCATTTCCAT	501	94°C, 60s; 57°C, 45s; 72°C, 60: 30 cycles	(Timsit et al., 2020)
***OXA_-24-like_***	GGTTAGTTGGCCCCCTTAAA AGTTGAGCGAAAAGGGGATT	246		
***OXA_-58-lik_***	AAGTATTGGGGCTTGTGCTG CCCCTCTGCGCTCTACATAC	599		

*Before the first cycle of gene amplification, the sample was subjected to denaturation at 94°C for a duration of 5 minutes. Following the final cycle, the sample underwent an extension phase at 72°C for 5 minutes.

### Identification of global clones

Alleles of *ompA*, *csuE* and *OXA_-51-like_* genes were amplified using multiplex-PCR method in order to identify group 1 (GC2) or group 2 (GC1) as previously described (Turton et al., 2007). The primers sequences are provided in **Table 2**. Each PCR reaction mixture contained: 12.5 μL PCR Master Mix (Ampliqon, Denmark), which contains Taq DNA polymerase, PCR Buffer, and dNTPs, 1 μL of 20 pM of each primer (Metabion, Germany) and 100 ng of genomic DNA. Amplification reaction was performed by thermal cycler with an initial denaturation at 95°C for 5 min, followed by 30 cycles of denaturation at 95°C for 45s, annealing at 57°C for 45s, and extension at 72°C for 1 min, afterwards final extension at 72°C for 5 min. After visualization of products by gel electrophoresis, if all three genes (**Table 2**) associated with group I are detected as positive, the isolates are classified under group 1. Similarly, if all three genes associated with group II are positive, the isolates are classified under group 2. In cases when *ompA* from group II, along with *csuE* and *OXA66* from group I, are positive, the isolates are classified under group 3 of the global clone.

**Table 2:** Primers used for identification of Global Clones.

*Gene*	Primer sequence (5' to 3')	Product size(bp)	Tm (°C)	Ref
*Group1ompA Forward*	GATGGCGTAAATCGTGGTA	355	55	(Chen and Huang, 2013)
*Group 1ompA Reverse*	CAACTTTAGCGATTTCTGG	355	53	
*Group 1csuE Forward*	CTTTAGCAAACATGACCTACC	702	57	
*Group 1csuE Reverse*	TACACCCGGGTTAATCGT	702	54	
*Group1OXA66 Forward*	GCGCTTCAAAATCTGATGTA	559	54	
*Group1OXA66 Reverse*	GCGTATATTTTGTTTCCATTC	559	54	
*Group 2ompA Forward*	GACCTTTCTTATCACAACGA	343	54	
*Group 2ompA Reverse*	CAACTTTAGCGATTTCTGG	343	57	
*Group 2csuE Forward*	GGCGAACATGACCTATTT	580	52	
*Group 2csuE Reverse*	CTTCATGGCTCGTTGGTT	580	54	
*Group2OXA69 Forward*	CATCAAGGTCAAACTCAA	162	49	
*Group2OXA69 Reverse*	TAGCCTTTTTTCCCCATC	162	52	

### Repetitive Extragenic Palindromic Element PCR (Rep-PCR) Genotyping

*A.baumannii* isolates were subjected to molecular typing by REP-PCR using the primer pairs REP1R-I (IIIICGICGICATCIGGC) and REP 2-I (ICGICTTATCIGGCCTAC) which were implemented to amplify putative REP like elements in the bacterial chromosomes (Chen and Huang, 2013). DNA amplification was performed in a final volume of 25 μl containing 12.5 μl of 2X Multi-Star PCR Master Mix (Bio-FACT, South Korea), 1 μl of each primer, 8.5 μl of distilled water, and 2 μl of the template DNA. Amplification reaction was performed by thermal cycler with an initial denaturation at 95°C for 10 min, followed by 35 cycles of denaturation at 95°C for 1 min, annealing at 45°C for 1 min, and extension at 72°C for 1 min, afterwards final extension at 72°C for 16 min. After visualization of products by gel electrophoresis, REP-PCR patterns were analyzed Using http://insilico.ehu.es/dice_upgma/ and isolates that revealed similarity cut-off ≥80% of their banding patterns were considered as the same types and isolates with similarity cut-off <80% were taken as different types.

### Statistical analysis

All data regarding the results of microbial tests, clinical findings and demographic characteristics, were added to the statistical package IBM SPSS Version 26 (Armonk, NY, USA) and analysis was performed using descriptive statistical tests.

## Results

### Prevalence of *A. baumannii* infections in Patients with COVID-19

Between April and November 2021, a total of 3,868 patients were admitted to intensive care units (ICUs), of whom 528 were diagnosed with PCR-confirmed COVID-19. Among these, 8.1% (43/528) were infected with *A. baumannii* and were included in this study. All isolates were confirmed to be *A. baumannii* by amplifying the *gltA* gene. Of 43 A*. baumannii* isolates, 81.4% (35/43) were recovered from respiratory samples, 14% (6/43) from blood, and 4.6% (2/43) from urine samples. The majority of isolates were recovered from males, comprising 62.8% (27/43). The age range was 18 to 87 years, with a mean age of 66.7 years. The most prevalent comorbidity in patients was hypertension, affecting 48.8% (21/43), followed by chronic heart disease in 39.5% (17/43), and diabetes in 30.2% (13/43). Ultimately, at the end of a median length of stay of 26.2 days, 88.4% (38/43) patients had died (**Table 3**). Meropenem was administered to all patients in the current study. Additionally, 91% (39/43) of the patients received a combination therapy of meropenem and colistin.

**Table 3:** Demographic Characteristics and laboratory findings of Hospitalized COVID-19 Patients (n=43) with *A. baumannii* coinfection.

Parameters		(Mean ± SD)
**Age**		66.7 *±* 11.1
**Duration of Hospital Stay**		26.2 *±* 27/1
**Laboratory findings on admission**	CRP (mg/L)	110 *±* 42.6
	ESR	89 *±* 36.3
	WBC	11800 *±* 19591
	Platelet count	164 *±* 102
		**n (%)**
**Gender**	Male	27 (62.8)
	Female	16 (37.2)
**Outcome**	Expired	38 (88.4)
	Discharged	5 (11.6)
**Sample source**	Respiratory tract samples	35 (81.4)
	Blood	6 (14)
	Urine	2 (4.6)
**Comorbidities**	Diabetes	13 (30.23)
	Hypertension	21 (48/84)
	Chronic heart disease	17 (39.53)
	Chronic respiratory disease	1 (2.33)
	Immunodeficiency	7 (16.28)
	Cancer	4 (9.3)
	addiction	3 (6.98)
	Chronic kidney disease	5 (11.63)

### Antimicrobial susceptibility tests

All 43 A*. baumannii* isolates were tested against a panel of 11 antibiotic discs and colistin as recommended by the CLSI-2021. The frequency of resistance to most tested antibiotics was high. Among the 43 A*. baumannii* strains isolated from COVID-19 patients, the highest resistance rate was observed against cefotaxime 100% (43/43), followed by 97.7% (42/43) resistance against each imipenem, meropenem, piperacillin-tazobactam and ciprofloxacin. The resistance rate against cefepime and ampicillin-sulbactam was 95.3% (41/43), 93% (40/43) against gentamicin, 90.7% (39/43) against each trimethoprim-sulfamethoxazole and amikacin and 88.4% (38/43) against ceftazidime. The most active antimicrobial agent against *A. baumannii* strains from COVID-19 patients was colistin with 55.8% (24/43) sensitivity. Most of the isolates, 91% (39/43), were XDR (resistant to +3 antibiotic classes), and 9% (4/43) were MDR phenotypes. Ten distinct patterns of antibiotic resistance were identified among 43 A*. baumannii* strains from COVID-19 patients in which the most prevalent patterns were resistant to all tested antibiotics expect colistin 44.2% (19/43) and resistant to all tested antibiotics 34.8% (15/43), respectively (**Table 4**).

**Table 4:** The demographic, molecular characteristics and antimicrobial resistance profile of 43 *Acinetobacter baumannii* isolates obtained from patients with Covid-19 infection.

Isolate No.	Sample source	Gender	Outcome	Resistance pattern	Resistance gene	REP type
***AB2***	Teracheal discharge	Male	Expired	IMI, MEM, CTX, CAZ, CIP, GM, AK, TS, SAM, PTZ, CPM	*aac6^´^Ib, VIM, TEM*	G
***AB3***	Teracheal discharge	Female	Expired	IMI, MEM, CTX, CAZ, CIP, GM, AK, TS, SAM, PTZ, CPM	*aac6^´^Ib, OXA_-23-Like_*	Untypeable
***AB5***	Teracheal discharge	Male	Expired	IMI, MEM, CTX, CAZ, CIP, GM, AK, TS, SAM, COL, PTZ, CPM	*aac6^´^Ib*	G
***AB6***	BAL	Male	Expired	IMI, MEM, SAM, TS, CTX	*aac3-Ia, OXA_-23-Like_*	D
***AB7***	Teracheal discharge	Male	Expired	IMI, MEM, CTX, CAZ, CIP, GM, TS, SAM, PTZ, CPM	*aac6^´^Ib, NDM, VIM, KPC, TEM, OXA_-23-Like_*	F
***AB8***	Urine	Male	Expired	IMI, MEM, CTX, CAZ, CIP, GM, AK, TS, SAM, PTZ, CPM, COL	*aac6^´^Ib, NDM, TEM, OXA_-23-Like_*	C
***AB12***	Teracheal discharge	Female	Expired	IMI, MEM, CTX, CIP, GM, AK, SAM, COL, PTZ, CPM	*aph3´Ia, ant2´ Ia, OXA_-23-Like,_ OXA_-58-Like_*	Untypeable
***AB19***	Teracheal discharge	Male	Expired	IMI, MEM, CTX, CAZ, CIP, GM, AK, TS, SAM, PTZ, CPM	*aac6^´^Ib, OXA_-23-Like_*	G
***AB24***	Teracheal discharge	Male	Expired	IMI, MEM, CTX, CAZ, CIP, GM, AK, TS, SAM, COL, PTZ, CPM	*aac6^´^Ib, NDM, VIM, KPC*	H
***AB28***	Teracheal discharge	Female	Expired	IMI, MEM, CTX, CAZ, CIP, GM, AK, TS, SAM, PTZ, CPM	*aac6^´^Ib, OXA_-24-Like_*	G
***AB33***	Blood	Female	Expired	IMI, MEM, CTX, CAZ, CIP, GM, AK, TS, SAM, PTZ, CPM	*aac6^´^Ib, ant2´ Ia, TEM, OXA_-23-Like_*	J
***AB34***	Teracheal discharge	Male	Expired	IMI, MEM, CTX, CAZ, CIP, GM, AK, TS, SAM, PTZ, CPM	*aac6^´^Ib, ant2´ Ia, OXA_-23-Like_*	A
***AB36***	Teracheal discharge	Female	Expired	IMI, MEM, CTX, CAZ, CIP, GM, AK, TS, SAM, PTZ, CPM	*aac6^´^Ib, ant2´ Ia, aph3´Ia, NDM, OXA_-23-Like_*	G
***AB37***	Teracheal discharge	Female	Expired	IMI, MEM, CTX, CAZ, CIP, GM, AK, TS, SAM, PTZ, CPM	*aac6^´^Ib, aac3-Ia, aph3´Ia, NDM, OXA_-23-Like_*	G
***AB39***	Teracheal discharge	Male	Discharged	IMI, MEM, CTX, CAZ, CIP, GM, AK, TS, SAM, COL, PTZ, CPM	*aac6^´^Ib, ant2´ Ia, NDM, TEM, OXA_-23-Like,_ OXA_-58-Like_*	G
***AB42***	Teracheal discharge	Male	Expired	IMI, MEM, CTX, CAZ, CIP, GM, AK, TS, SAM, COL, PTZ, CPM	*OXA_-24-Like_*	G
***AB47***	Teracheal discharge	Female	Discharged	IMI, MEM, CTX, CAZ, CIP, GM, AK, TS, SAM, PTZ, CPM	*aac6´Ib, ant2´ Ia, OXA-23-Like*	B
***AB48***	blood	Male	Discharged	IMI, MEM, CTX, CAZ, CIP, GM, AK, TS, SAM, PTZ, CPM	*aac6´Ib, ant2´ Ia, OXA-23-Like, NDM*	G
***AB50***	Teracheal discharge	Male	Expired	IMI, MEM, CTX, CAZ, CIP, GM, AK, SAM, PTZ, CPM	*aac6´Ib, ant2´ Ia, OXA-24-Like*	G
***AB51***	Blood	Male	Expired	IMI, MEM, PTZ, CIP, CPM, GM, CTX, AK	*aac6´Ib, ant2´ Ia, OXA-24-Like*	G
***AB53***	Teracheal discharge	Male	Expired	IMI, MEM, PTZ, CIP, CPM, GM, CTX, AK	*aac6^´^Ib, aac3-Ia*	G
***AB57***	Teracheal discharge	Male	Expired	IMI, MEM, CTX, CAZ, CIP, GM, AK, TS, SAM, PTZ, CPM	*VIM*	G
***AB58***	Teracheal discharge	Male	Expired	IMI, MEM, CTX, CAZ, CIP, GM, AK, TS, SAM, COL, PTZ, CPM	*aac6^´^Ib, aph3´Ia, NDM, TEM*	G
***AB59***	Blood	Female	Expired	IMI, MEM, CTX, CIP, GM, AK, TS, SAM, COL, PTZ, CPM	*OXA-48, aac6^´^Ib*	G
***AB60***	Teracheal discharge	Male	Expired	IMI, MEM, CTX, CAZ, CIP, GM, AK, TS, SAM, PTZ, CPM	*aac6´Ib, KPC, TEM, OXA-23-Like*	G
***AB62***	Teracheal discharge	Male	Expired	IMI, MEM, CTX, CAZ, CIP, GM, AK, TS, SAM, COL, PTZ, CPM	*aac6^´^Ib, aph3´Ia, TEM, OXA-23-Like*	G
***AB68***	Teracheal discharge	Female	Expired	IMI, MEM, CTX, CAZ, CIP, GM, AK, TS, SAM, COL, PTZ, CPM	*ant2´ Ia, aph3´Ia, NDM, VIM, OXA-24-Like*	E
***AB69***	Teracheal discharge	Male	Expired	IMI, MEM, CTX, CAZ, CIP, GM, AK, TS, SAM, PTZ, CPM	*NDM, aac6^´^Ib*,	G
***AB72***	Teracheal discharge	Male	Expired	IMI, MEM, CTX, CAZ, CIP, GM, AK, TS, SAM, PTZ, CPM	*aac6^´^Ib, TEM, OXA-24-Like*	I
***AB75***	Teracheal discharge	Female	Expired	IMI, MEM, CTX, CAZ, CIP, GM, AK, TS, SAM, PTZ, CPM	*TEM*	K
***AB76***	Blood	Female	Expired	IMI, MEM, CTX, CAZ, CIP, GM, AK, TS, SAM, PTZ, CPM	*aph3´Ia, TEM, OXA-23-Like, OXA-24-Like*	G
***AB78***	Teracheal discharge	Female	Expired	IMI, MEM, CTX, CAZ, CIP, GM, AK, TS, SAM, COL, PTZ, CPM	*ant2´ Ia, IMP*	L
***AB79***	Teracheal discharge	Female	Expired	IMI, MEM, CTX, CAZ, CIP, GM, AK, TS, SAM, COL, PTZ, CPM	*ant2´ Ia, OXA-23-Like*	Untypeable
***AB80***	BAL	Male	Expired	IMI, MEM, CTX, CAZ, CIP, GM, AK, TS, SAM, COL, PTZ, CPM	*ant2´ Ia, TEM, OXA-23-Like*	G
***AB84***	Teracheal discharge	Male	Expired	IMI, MEM, CTX, CAZ, CIP, GM, AK, TS, SAM, COL, PTZ, CPM	*TEM, OXA-24-Like*	G
***AB85***	Teracheal discharge	Male	Expired	IMI, MEM, CTX, CAZ, CIP, GM, AK, TS, SAM, PTZ, CPM	*ant2´ Ia, TEM, OXA-23-Like*	G
***AB86***	Teracheal discharge	Female	Expired	IMI, MEM, CTX, CAZ, CIP, GM, AK, TS, SAM, PTZ, CPM	*ant2´ Ia, TEM, OXA-23-Like*	G
***AB89***	Teracheal discharge	Male	Expired	IMI, MEM, CTX, CAZ, CIP, GM, AK, TS, SAM, COL, PTZ, CPM	*NDM, TEM, OXA-24-Like*	G
***AB96***	Teracheal discharge	Female	Expired	IMI, MEM, CTX, CAZ, CIP, GM, AK, TS, SAM, COL, PTZ, CPM	*ant2´ Ia, TEM, OXA-24-Like*	G
***AB98***	Teracheal discharge	Male	Discharged	IMI, MEM, CTX, CAZ, CIP, TS, SAM, COL, PTZ, CPM	*aac6^´^Ib, TEM, OXA-23-Like*	Untypeable
***AB105***	Teracheal discharge	Male	Expired	IMI, MEM, CTX, CAZ, CIP, GM, AK, TS, SAM, COL, PTZ, CPM	*aac6^´^Ib, ant2´ Ia, TEM, OXA-24-Like*	G
***AB109***	Teracheal discharge	Male	Expired	PTZ, SAM, CIP, TS, CAZ, CTX	*aac6^´^Ib, OXA-58-Like*	G
***AB118***	Wound	Female	Discharged	IMI, MEM, CTX, CAZ, CIP, GM, AK, TS, SAM, COL, PTZ, CPM	*OXA-24-Like*	G

BAL, Broncho Alveolar Lavage; IMI, Imipenem; MEM, Meropenem; PTZ, Piperacillin-tazobactam; SAM, Ampicillin-sulbactam; CTX, Cefotaxime; CAZ, Ceftazidime; CIP, Ciprofloxacin; GM, Gentamicin; AK, Amikacin; TS, Trimethoprim-sulfamethoxazole; COL, Colistin; CPM, Cef.

### Detection of ARGs

The rate of genes encoding class D carbapenemases which were detected using Multiplex PCR was *OXA_-23-like_* 67.4% (29/43), *OXA_-24-like_* 30.2% (13/43) and *OXA_-58-like_* 7% (3/43). Amongst the MBLs; *bla*_NDM_ was detected in 41.8%, (18/43), *bla*_IMP_ 2.3% (1/43) and *bla*_VIM-1_ 9.3% (4/43) isolates. Class A β-lactamase *bla*_KPC_ was detected in 9.3% (4/43) isolates and 48/8% (21/43) of isolates harbored *bla*_TEM_ gene. The following percentages of selected genes encoding aminoglycoside-modifying enzymes (AMEs) [*aac(6’)-Ib* 65.1% (28/43)*, aac(3)-Ia*, 4.7% (2/43)*, ant(2`)-Ia* 46.5% (20/43), *aph(3’)-Ia* 16.3% (7/43)] were observed among analyzed strains. PCR amplification of *mcr-1* primers did not bring about a product for colistin resistance encoding gene. Our study shown the presence of at least one AME and β-lactamase encoding gene in 88.37% (38/43) and 67.4% (29/43) of isolates, respectively (**Table 4**).

### Global clones lineages

**Figure 1:**
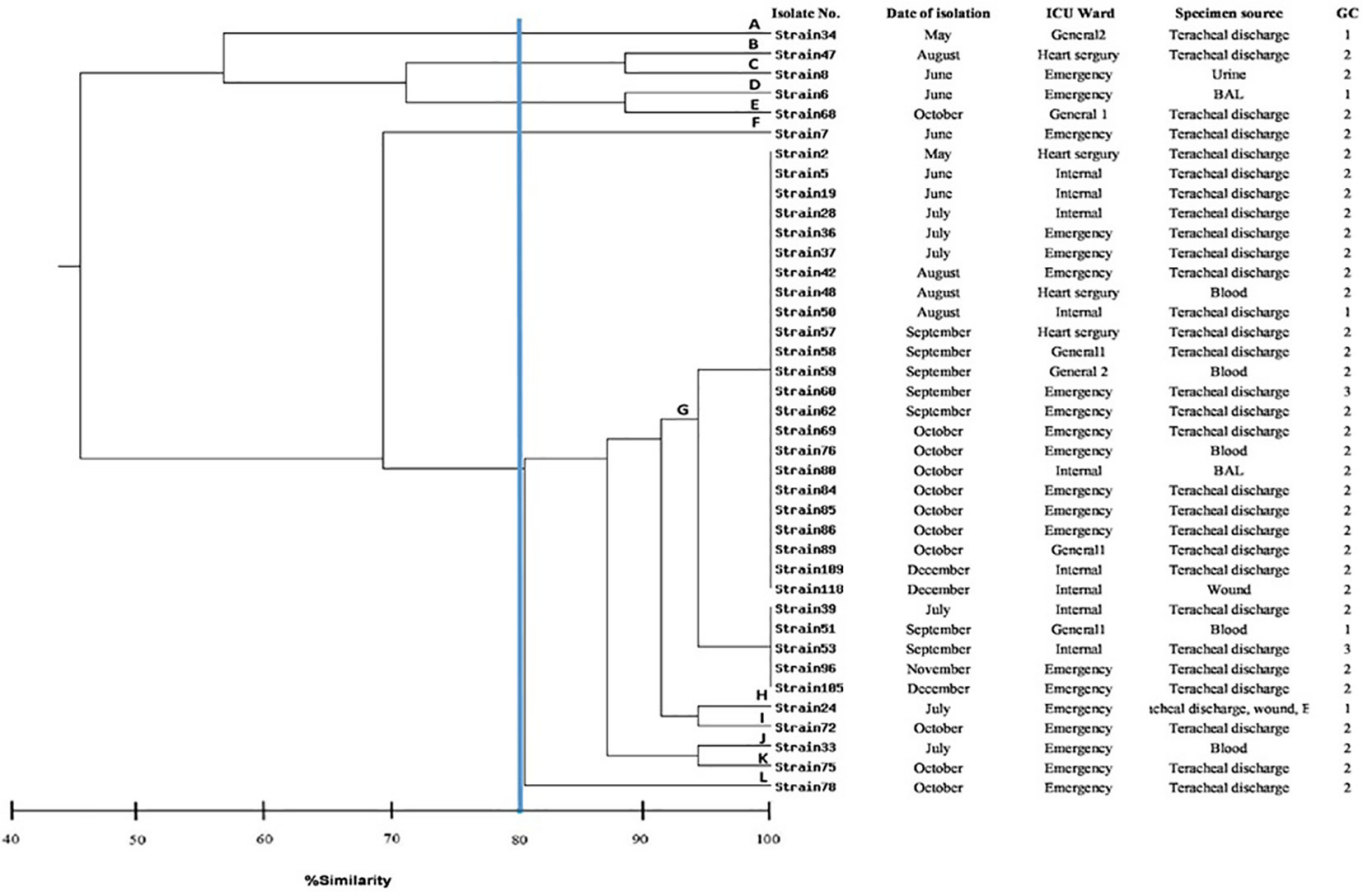
Dendrogram showing the genetic relatedness of 39 typeable strains of *Acinetobacter baumannii* determined by REP-PCR analysis using the Dice similarity coefficient. The vertical line displays the 80% similarity cut-off value. Based on a similarity index ≥80%, 12 genotypes were found. Each genotype were labelled A to L. Numbers at the terminal branches are strain name.

Multiplex PCR for the identification of GCs indicated 83.7% (36/43), 11.6% (5/43) and 4.7% (2/43) of *A. baumannii* isolates belonged to GC II, GC I and GC III, respectively (**Figure 1**).

### REP-PCR genotyping

The clonal relatedness of 43 A*. baumannii* isolates was studied by REP-PCR, which amplified 5 to 9 bands with molecular weight ranging from 150 bp to 1.2 kb and were labelled A to L. Among 43 A*. baumannii* isolates, 39 isolates were typed by REP-PCR and no bands were observed in 4 isolates (**Figure 1**). By using a cut off value of ≥80% as the threshold, 12 patterns among 39 tested isolates were observed, in which 11 patterns were detected once, while the remaining 1 pattern was repeatedly observed. Genotype G was the most prevalent, accounting for 72% (28/39) of the isolates. Of this genotype, 96% (27/28) exhibited resistance to carbapenems.

## Discussion

Given the increased incidence of infections caused by MDR and XDR strains of *A. baumannii*, this pathogen has emerged as a significant threat to vulnerable ICU patients, especially those facing critical conditions, such as COVID-19 patients. In this cross-sectional study, the prevalence of *A. baumannii* co-infection in COVID-19 patients was 8.1%, which was similar with other reports from Iran (7.44%) (Abdollahi et al., 2021), Jordan (8.1%) (Alsheikh et al., 2022) and India (8.9%) (Khurana et al., 2021); whereas, in other studies conducted in Italy (15.5%) (Cultrera et al., 2021), China (21.8%) (Sang et al., 2021), Iran (41% and 51%) (Jamnani et al., 2022; Pourajam et al., 2022) and United States (62%) (Perez et al., 2020), the incidence of co-infection was significantly higher than the rate of present Study. The observed variation may be attributed to the differences in healthcare systems and infection control practices (Organization WH, 2016), patient demographics and characteristics (Grasselli et al., 2020b), diagnostic methods and surveillance (Antibiotic resistance threats in the United States, 2019, 2019), regional epidemiology (Peleg et al., 2008) and study design and methodology (von Elm et al., 2007). Our study reveals that the number of males infected with *A. baumannii* in ICU was higher than the number of females infected with *A. baumannii*. This may be due to the differences in immunologic reaction because testosterone has immunosuppressive effect in males while estradiol has a pro-inflammatory effect in females (Hafiz et al., 2023). The mortality rates among COVID-19 patients varied widely across different countries, ranging from 16% to 100% (Grasselli et al., 2020a; Novović et al., 2023). Our study revealed a high mortality rate (88.4%) among COVID-19 patients infected with *A. baumannii*. A study conducted in Qom, Iran, Sharifipour et al. observed that among 17 COVID-19 patients with *A. baumannii* infections, 17 (100%) died. In another study in Serbia showed the mortality rate was 100% (Novović et al., 2023), while a study in South Korea had a lower mortality rate of 64.3% (Kim et al., 2023). Possible explanations for these differences in mortality rates might be attributed to variation in clinical supervision, the accessibility of therapeutics, healthcare organization and staff training (Malaeb et al., 2023). Additionally, comorbidities also play a crucial role in the mortality of COVID-19 patients. Hypertension, cardiovascular disease, and diabetes were the most common comorbidities observed in our study, consistent with similar findings elsewhere (Woodford et al., 2006; Abdollahi et al., 2021). In this study, the median length of stay in the ICU was higher (26.2 days) compared with other studies from Iran (15.8 days), South Korea (16.8 days) and Italy (12 days) (Grasselli et al., 2020a). The variation in the median length of stay might be related to several factors, including differences in healthcare systems, treatment protocols, patient demographics, and the prevalence and resistance patterns of *A. baumannii* (Vincent et al., 2014; Rosenthal et al., 2020). Regarding antibiotic resistance, our isolates were extremely resistant to the most evaluated antibiotics. Furthermore, out of the 43 isolates, 98% were CRAB, 91% were XDR and 9% were MDR. This high rate of antibiotic resistance in our hospital was possible as a result of the excessive or misuse of antibiotics, which creates the selection pressure for development of resistance. Alarmingly, 44.2% of our isolates were resistant to colistin, that is considered to be high with respect to previous studies (Boral et al., 2019; Ibrahim, 2019; Hafiz et al., 2023). In spite of high resistance rate to colistin, none of the isolates in present study harbored *mcr-1* gene. It seems that other colistin resistance mechanisms reported in *A. baumannii*, including mutation of the PmrAB system and loss of lipopolysaccharide (LPS) production might be involved in the resistance (Palmieri et al., 2020; Zafer et al., 2023). In this study, we identified various ARGs, and the most were *OXA_-23-like_* (67.4%), *aac(6’)-Ib* (65.1%), *bla*_TEM_ (48.8%), *ant(2`)-Ia* (46.5%), *bla*_NDM_ (41.8%) and *OXA_-24-like_* (30.2%). In a previous study from the southwestern of Iran, Farajzadeh et al. observed that *OXA_-23-like_* (32.2%), *bla*_VIM_ (31.4%), *bla*_IMP_ (25.7%) were the most common carbapenemase genes among clinical isolates of *A. baumannii* (Farajzadeh Sheikh et al., 2020). In Egypt, Benmahmod et al. reported that *OXA_-23-like_* (94%), *bla*_KPC_ (56%) and *bla*_NDM_ (30%) were the most prevalent carbapenemase genes among *A. baumannii* strains isolated from clinical samples (Benmahmod et al., 2019). In Saudi Arabia, Alyamani et al. observed that *OXA_-23-like_* (91%) and *bla*_TEM_ (71%) were the most prevalent carbapenemase and class A β-lactamase encoding genes among *A. baumannii* isolates (Alyamani et al., 2015). In a Chinese study, 100%, 100%, 67.53% and 31.17% of the CRAB harbored *bla*_VIM_, *OXA_-23-like_*, *bla*_IMP_ and *bla*_NDM_ genes, respectively (Zhu et al., 2022). In Algeria, the most prevalent AME genes in *A. baumannii* isolates were *aac(3)-Ia* (91.1%) and *aph(3′)-VI* (50.7%) (Bakour et al., 2013). A study from China, Nie et al. reported that *ant(3′′)-I* (66.47%), *aac(3)-I* (45.09%), *aph(3′)-I* (34.1%) and *aac(6’)-Ib* (32.37%) were the most prevalent AME genes among *A. baumannii* isolates (Nie et al., 2014). As mentioned earlier, the prevalence of ARGs varied widely between different countries. These differences may be attributed to different patterns in use of antimicrobial agents, horizontal spread of resistance determinants, dissemination of specific clones harboring various types of ARGs and the number of studied isolates. In the current study, 8 (18.6%) isolates were negative for the seven carbapenemases tested, indicating that other mechanisms such as porin loss, overexpression of efflux pumps, AmpC enzymes might contribute to carbapenem resistance (may lead to resistance to carbapenems), which were not investigated in present study (Cai et al., 2019). Our data exhibited the coexistence of carbapenemases and aminoglycoside resistance genes among CRAB isolates, similar to previous reports from India (Karthikeyan et al., 2010) and Iran (Beig et al., 2023). These findings highlighted the difficulty in treating CRAB due to the presence of multiple ARGs. Infections with CRAB carrying multiple ARGs are usually associated with a high level of mortality and morbidity. As reported previously, the majority of *A. baumannii* isolates that are MDR and XDR belong to two international clones (GC1 and GC2) (Hamed et al., 2022). In agreement with other studies (Al-Sultan et al., 2015; Abdollahi et al., 2021), the current study demonstrated the predominance of GC2 isolates in our collection (83.7%). Molecular typing is a relevant tool for epidemiological purposes and examining the genetic structure of the organisms (Hallin et al., 2012). To further examine the genetic relatedness of *A. baumannii* isolates, the REP-PCR typing method was employed. In this work, 43 isolates were examined, leading to the identification of 12 distinct patterns among the 39 typeable isolates. Notably, a significant proportion (72%) of our isolates were grouped into a single genotype or clone, suggesting that these isolates were closely related and the spread of these isolates were associated with a clonal outbreak. It should be noted that we observed that isolates belonging to genotype G have the distinct ARGs, indicating that the horizontal transmission may occurred. Generally, isolation of many resistant bacteria in hospitals such as CRAB can be driven by two epidemiological scenarios: the emergence and spread of a specific clone, or the persistence and coexistence of multiple clones (Al-Sultan et al., 2015). Our data are in concordance with the first scenario, because one clone is circulating in our hospital. To prevent the circulation of specific clones in hospitals, effective infection prevention and control are crucial. The hospital infection control committee (HICC) is present in the majority of Iranian hospitals, but HICC lacks an efficient program for infection prevention and control (Emaneini et al., 2018; Esfandiari et al., 2018). These committees exist generally on paper; in practice, they scarcely exist (Mamishi et al., 2014; Emaneini et al., 2018; Esfandiari et al., 2018). These issues are exacerbated during crises like the COVID-19 pandemic, potentially facilitating the horizontal transmission and spread of specific clones of multidrug-resistant organisms (Endale et al., 2023). It should be emphasized that the present study had several limitations. First, the major limitation of this study was its retrospective design. Second, owing to the small sample size and single-center design, the findings may not be generalizable to patient populations in different hospitals and countries. Third, utilization other typing approaches such as multi-locus sequence typing and pulsed-field gel electrophoresis for further genotypic characterization is recommended.

## Conclusion

The high prevalence of MDR *A. baumannii* such as carbapenem and colistin-resistant strains, poses a significant concern for the treatment of COVID-19 patients, heightening the risk of therapeutic failure. The REP-PCR typing data demonstrate the dissemination of a single *A. baumannii* clone carrying multiple ARGs within our hospital. Regarding the limited therapeutic options, it is crucial to implement effective prevention and containment policies to curb the spread of these isolates.

## Data Availability

The raw data supporting the conclusions of this article will be made available by the authors, without undue reservation.
